# Influence of Ammonium Ions, Organic Load and Flow Rate on the UV/Chlorine AOP Applied to Effluent of a Wastewater Treatment Plant at Pilot Scale

**DOI:** 10.3390/ijerph15061276

**Published:** 2018-06-16

**Authors:** Eduard Rott, Bertram Kuch, Claudia Lange, Philipp Richter, Ralf Minke

**Affiliations:** Institute for Sanitary Engineering, Water Quality and Solid Waste Management, University of Stuttgart, Bandtäle 2, 70569 Stuttgart, Germany; bertram.kuch@iswa.uni-stuttgart.de (B.K.); claudia.lange@dekra.com (C.L.); philipp.richter@iswa.uni-stuttgart.de (P.R.); ralf.minke@iswa.uni-stuttgart.de (R.M.)

**Keywords:** ammonium, emerging contaminants, pilot plant, UV/chlorine AOP, UV/HOCl, wastewater treatment

## Abstract

This work investigates the influence of ammonium ions and the organic load (chemical oxygen demand (COD)) on the UV/chlorine AOP regarding the maintenance of free available chlorine (FAC) and elimination of 16 emerging contaminants (ECs) from wastewater treatment plant effluent (WWTE) at pilot scale (UV chamber at 0.4 kW). COD inhibited the FAC maintenance in the UV chamber influent at a ratio of 0.16 mg FAC per mg COD (*k*_HOCl–COD_ = 182 M^−1^s^−1^). An increase in ammonium ion concentration led to a stoichiometric decrease of the FAC concentration in the UV chamber influent. Especially in cold seasons due to insufficient nitrification, the ammonium ion concentration in WWTE can become so high that it becomes impossible to achieve sufficiently high FAC concentrations in the UV chamber influent. For all ECs, the elimination effect by the UV/combined Cl_2_ AOP (UV/CC) was not significantly higher than that by sole UV treatment. Accordingly, the UV/chlorine AOP is very sensitive and loses its effectiveness drastically as soon as there is no FAC but only CC in the UV chamber influent. Therefore, within the electrical energy consumption range tested (0.13–1 kWh/m^3^), a stable EC elimination performance of the UV/chlorine AOP cannot be maintained throughout the year.

## 1. Introduction

The prevention of the emission of anthropogenic emerging contaminants (ECs) in surface waters is becoming increasingly important, as such compounds can be endocrine disrupting [1] and carcinogenic [2]. Since these compounds are mainly introduced into the environment via wastewater treatment plant effluents (WWTE), additional treatment steps, such as the advanced oxidation process (AOP), are provided for in wastewater treatment plants (WWTPs). One such method is the UV/chlorine AOP. The principle of this process is the transformation of free available chlorine (FAC), e.g., in the form of hypochlorous acid (HOCl) or the hypochlorite anion (OCl^−^) (pK_a_ = 7.5), by UV radiation into highly reactive radicals (Equations (1)–(3)), with the aim of oxidizing the ECs to CO_2_ and H_2_O or at least rendering them biodegradable [3,4,5,6]:

HOCl + UV photons → •OH + Cl•(1)

ClO^−^ + UV photons → •O^−^ + Cl• (2)

•O^−^ + H_2_O → •OH + OH^−^ (3)

In a study with real effluent of a WWTP (continuous operation with 1 m^3^/h, medium pressure UV lamp operated at 0.4–1.0 kW) by Rott et al. [7] it was shown at pilot scale that the UV/chlorine AOP is superior to the UV/H_2_O_2_ AOP [8] in terms of the elimination of ECs, the bacterial count and the total estrogenic activity, as much lower mass concentrations of oxidant are required. All investigations in this study were carried out at NH_4_^+^-N concentrations <0.1 mg/L.

A major concern associated with the UV/chlorine AOP is the formation of potentially toxic and lipophilic halogenated degradation by-products such as adsorbable organohalogens (AOX) [7,9,10]. Side reactions contribute to the fact that the dosed chlorine immediately reacts to form combined chlorine (CC) or decomposes into chloride. Ammonium ions belong to the most important compounds in WWTE making it difficult to maintain free chlorine in such a form of wastewater. For example, chlorine reacts with ammonium ions preferably to form chloramines, as shown in Equation (4) in the form of monochloramine [11]:

HOCl + NH_4_^+^ → NH_2_Cl + H_2_O + H^+^ (4)

**Figure 1:**
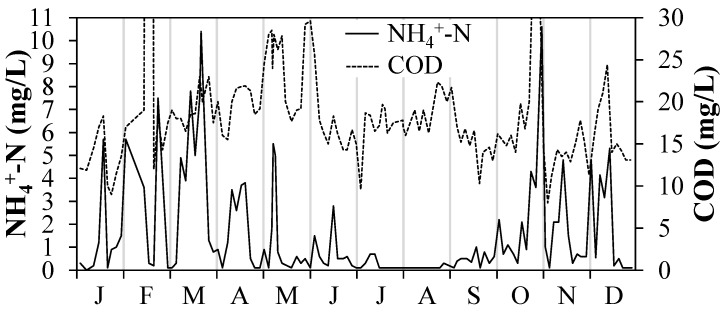
Ammonium ions and chemical oxygen demand (COD) concentrations determined in WWTE of the LFKW (x-axis: months).

An important task of WWTPs is the removal of nitrogen. Ammonium ions in the feed are oxidized to nitrate (NO_3_^−^) by aerobic, autotrophic, nitrifying microorganisms. This, in turn, is converted by predominantly heterotrophic, denitrifying bacteria under anaerobic conditions to gaseous, elementary nitrogen (N_2_), which escapes into the atmosphere. The speed of both processes is severely impaired at low temperatures and comes to a standstill at 8 °C. In the temperate zone, where very cold and warm seasons alternate, the elimination of ammonium ions in WWTPs is thus not guaranteed throughout. Figure 1 shows the ammonium ion concentration in the effluent of the WWTP (Lehr- und Forschungsklärwerk, LFKW, Stuttgart, Germany), the effluent of which was used in the investigations by Rott et al. [7] and this work. It becomes clear that NH_4_^+^-N concentrations of up to 10 mg/L can occur in cold seasons. In this year, the wastewater temperature varied between 8 and 21 °C, with 125 days of it being <13 °C. It is therefore necessary to find out to what extent the UV/chlorine AOP is influenced by high NH_4_^+^ concentrations in the effluent of the WWTP when it is used for the elimination of ECs.

The formation of chloramines in the UV/chlorine process does not necessarily lead to the absence of oxidation of ECs. It is known that in the presence of UV radiation chloramines are also converted to reactive radicals (e.g., aminyl and chlorine radicals) (Equation (5)) [6,9]:

NH_2_Cl + UV photons → NH_2_• + Cl• (5)

Only a few studies investigating the UV/chlorine AOP involve high ammonium ion concentrations in their experiments [9,12,13]. Generally, these investigations are only based on synthetic wastewater on a laboratory scale often only simulating the organic carbon content by the dosage of a specific compound such as *tert*-butanol or citric acid and urea [14,15]. However, real conditions in WWTE can be completely different. The aim of this work is therefore to investigate the influence of ammonium ions and the organic load (COD, Figure 1) in wastewater under realistic conditions, i.e., at pilot scale in continuous operation with real WWTE.

The article discussed here is to be understood as a continuation of the article by Rott et al. [7]. In this article, experiments with the pilot plant used here were carried out with WWTE of negligible ammonium ion concentrations of the same WWTP.

## 2. Materials and Methods

### 2.1. Electrical Energy Consumption

The electrical energy consumption E (in kWh/m^3^, Equation (6) with electrical power input P (in kW) and the flow rate F of the pilot plant (in m^3^/h)) is correlated to the running costs of a flow-through plant and is therefore an important factor for the technical applicability of the process [14]. Moreover, assuming first-order kinetics, the electrical energy consumption per order of compound removal (E_EO_) can be calculated using Equation (7), where c_0_ is the initial concentration of the compound and c is the concentration of the compound after treatment [16]:

(6) $$E\;=\;\frac{P}{F}$$

(7) $$E_{\mathrm{EO}}\;=\;\frac{P}{F\times \log\left(\frac{c_{0}}{c}\right)}$$

### 2.2. Overview of Experiments

Four experiments were carried out. In these experiments, either tap water spiked with diclofenac and carbamazepine (these ECs are among the most frequently found pharmaceuticals in water bodies and they are ineffectively removed in WWTPs [17]) or the effluent of a WWTP was treated with a pilot plant equipped with a medium pressure UV chamber. The WWTE was examined for 16 different ECs. The individual parameters were varied as follows:

- Exp. A: Variation of NH_4_^+^-N concentration (0.5, 1.0, 1.5 mg/L) in spiked tap water (6.9 mg/L dosed free Cl_2_, 0.4 kW UV power, 1 m^3^/h flow rate)
- Exp. B: Variation of WWTE dilutions with spiked tap water (6.9 mg/L dosed free Cl_2_, 0.4 kW UV power, 1 m^3^/h flow rate)
- Exp. C: Variation of CC concentration (1–5 mg/L CC in UV chamber influent) on WWTE (0.0 and 0.4 kW UV power, 1 m^3^/h flow rate)
- Exp. D: Variation of flow rate (1, 2, 3 m^3^/h WWTE) at 3 mg/L FAC dosage in UV chamber influent and 0.4 kW UV power (0.13, 0.20, 0.40 kWh/m^3^ electrical energy consumption)

### 2.3. Chemicals and Reagents

NaOCl solution (14% active chlorine) and hydrochloric acid (HCl, 32%, AnalaR Normapur) were purchased from VWR International (Radnor, PA, USA). The used H_2_O_2_ solution (35%) provided by Siemens Water Technologies (Günzburg, Germany) was of technical grade. Carbamazepine (99%) and diclofenac sodium salt (>98.5%) were purchased from Sigma-Aldrich (St. Louis, MO, USA). NH_4_Cl (p.a.) was purchased from Merck KGaA (Darmstadt, Germany). Crystalline sodium thiosulfate pentahydrate (Na_2_S_2_O_3_∙5H_2_O, ≥99%) was purchased from Carl Roth (Karlsruhe, Germany). Dichloromethane (CH_2_Cl_2_, >99.8%) was purchased from Bernd Kraft GmbH (Duisburg, Germany). *N*,*N*-diethyl-*p*-phenylenediamine (DPD) was contained in powder pillows obtained from Hach (Berlin, Germany).

### 2.4. Tap Water (TW) and Wastewater Treatment Plant Effluent (WWTE)

The tap water used was analyzed for the following parameters the concentrations of which were mainly below the limit of detection: <0.1 mg/L NH_4_^+^-N, <5.0 mg/L COD, <1.5 mg/L DOC (dissolved organic carbon), 300–350 µS/cm electrical conductivity, <0.02 mg/L free Cl_2_, <0.02 mg/L total Cl_2_. Thus, the tap water had not been chlorinated in the waterworks when the experiments were conducted.

The municipal Treatment Plant for Education and Research (LFKW, Lehr- und Forschungsklärwerk) with a capacity of 30 L/s treats an average amount of 900,000 m^3^ per annum (9000 population equivalents). Its raw wastewater is composed of domestic wastewater and wastewater from the university grounds mainly of industrial effluents. After the primary clarifier, the wastewater is treated in separated denitrification and nitrification tanks (simultaneous P precipitation). The aerated sludge is separated in a secondary clarifier the effluent of which is additionally separated from particles by micro sieves (15–20 µm pore size).

**Figure 2:**
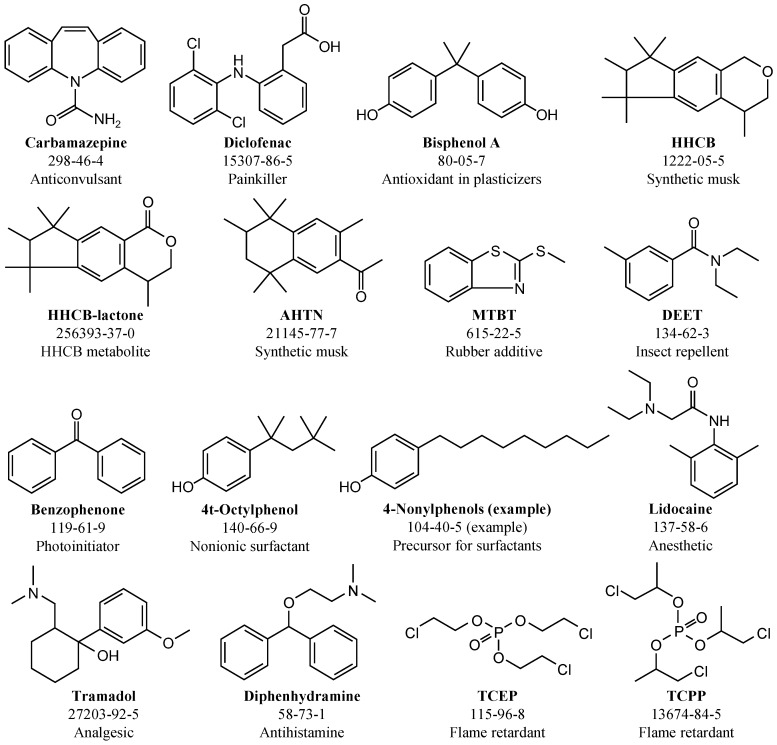
Emerging contaminants analyzed in WWTE samples with CAS numbers (Rott et al. [7] based on [18]).

In experiments with tap water and dilutions of WWTE, carbamazepine and diclofenac were spiked and analyzed. The initial concentrations of these compounds in raw samples of all experiments and important parameters characterizing the wastewater composition are given in Table 1. The temperature of the wastewater was between 14 and 19 °C. The pH varied slightly between 6.9 and 8.2. The WWTE was mainly composed of 5–8 mg/L DOC, approx. 20–30 mg/L COD and approx. 1000 µS/cm electrical conductivity. The NH_4_^+^-N concentration in WWTE could vary strongly between <0.1 and 6.7 mg/L (Table 1). In experiments with pure WWTE directly drained from the micro sieves effluent, fourteen other emerging contaminants presented in Figure 2 were analyzed. Their initial concentrations are given in the Supplementary Material (Table S1) and varied between 0.02 and 2.19 µg/L.

### 2.5. Pilot Plant

**Figure 3:**
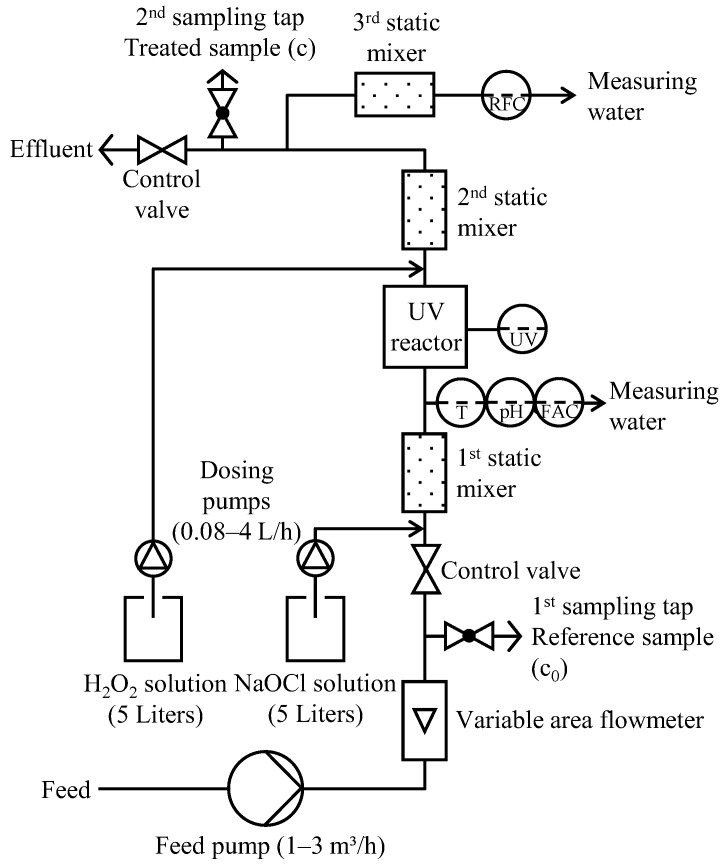
Technical scheme of the UV/chlorine AOP pilot plant (with changes from Rott et al. [7]).

The UV/AOP pilot plant (Figure 3, see Rott et al. [7] for more details) was placed next to the micro sieves of the WWTP. The plant was fed by means of an eccentric screw pump. The flow rate (1–3 m^3^/h) was controlled using a variable area flowmeter. A tap at the inlet of the plant allowed for the sampling of untreated sample (c_0_). NaOCl stock solution was dosed into the feed stream using a peristaltic pump (0.08–4 L/h). Subsequent to a static mixer, a portion of the feed stream was directed to a measuring cell where the temperature, pH (single junction, combination electrode sensor, Wallace & Tiernan, Günzburg, Germany) and free available Cl_2_ (FAC) (potentiostatic electrode amperometry sensor, Wallace & Tiernan, Günzburg, Germany) were analyzed. The medium pressure UV lamp (type: WTL 1000, 1 kW maximum power, 230 mm length × 22 mm diameter, Wallace & Tiernan, Günzburg, Germany), protected by a quartz sleeve with a thickness of 1 mm and cut-off at 200 nm wavelength, was installed in a stainless steel chamber (Wallace & Tiernan Barrier M35, 300 mm assembly dimension × 214 mm height × 600 mm length) (approx. contact time in the UV chamber: 6–10 s). The irradiance was visualized by a 4–20 mA UV sensor (signal in W/m^2^) on the cabinet. The UV chamber effluent could be mixed with H_2_O_2_ via a second peristaltic pump in order to quench residual free Cl_2_ (RFC). This study focused on the technical feasibility of the UV/chlorine process. Therefore, this peristaltic pump was mainly operated in automatic mode, i.e., the H_2_O_2_ dosage was automatically controlled by means of a chemical feed analyzer (via a further static mixer, a partial stream was passed into a further measuring cell where the RFC concentration could be measured) and process controller (MFC Analyzer/Controller) from Wallace & Tiernan. Since the FAC concentration could vary during an experiment while the H_2_O_2_ dosage was running and the experiments were limited in time, it was therefore not possible to determine the RFC concentration on a regular basis in case of missing H_2_O_2_ dosage. This aspect is therefore not addressed in this article. The contact time of the quenching agent from its dosage point to the effluent of the pilot plant was 4–6 s. The pilot plant effluent (treated sample (c)) could be sampled via a sampling tap at the outlet of the pilot plant (upper sampling tap). At a flow rate of 1 m^3^/h, the flow time in the pilot plant was 25–28 s.

### 2.6. Experimental Procedure

#### 2.6.1. Preparations

Prior to some experiments, carbamazepine, diclofenac and ammonium chloride (NH_4_Cl) were dissolved in pure water in separate 1 L flasks (stock solutions). In order to obtain similar carbamazepine and diclofenac concentrations in all spiked tap water experiments (Exp. A, B and C), at first two 1 L samples were collected from continuously circulated WWTE in a big tank (40 m^3^). In these samples, the concentrations of both compounds were analyzed. By adding a quantity of the abovementioned stock solutions matched to this concentration to 800 L of sample, an attempt was made to obtain similar initial EC concentrations in these experiments. As it can be seen in Table 1, only in some cases similar concentrations could not be achieved in all batches showing that an exact adjustment of the EC concentration in the µg/L range on this scale was challenging. This can be attributed to very fine residual pollution contaminated with the ECs in the 800 L sample tank despite meticulous cleaning of the tank prior to the experiment. Furthermore, the spiked compounds could adsorb on such deposits and the analyzed dissolved concentration of the ECs could therefore be lower than expected. However, previous experiments [7] had shown that as long as the initial concentrations of the ECs are in a similar range, comparable results of the c/c_0_-ratio can be determined.

#### 2.6.2. General Procedure

The feed pump (1 m^3^/h flow rate), UV lamp (operated at 0.4 kW), the NaOCl dosing pump and quenching agent dosing pump were switched on consecutively. After 10 min, the samples were collected. First two or three sample bottles (i.e., duplicate or triplicate samples) were filled with reference sample (c_0_). Subsequently, two or three sample bottles were filled with treated sample (c).

#### 2.6.3. Exp. A: Variation of NH_4_^+^-N Concentration in Spiked Tap Water

Four 800 L tap water batches with different concentrations of NH_4_^+^ were prepared (0.0, 0.5, 1.0 and 1.5 mg/L NH_4_^+^-N) and treated separately as described as follows. When the feed tank was filled with 800 L of tap water, the carbamazepine, diclofenac and NH_4_^+^ stock solutions were added to the tank. In order to achieve a good homogenization, the tank was stirred for 1 h. For each of the four batches, separately the general procedure described in Section 2.6.2 was performed (duplicate samples taken for the analysis). For all batches, the flow rate of the NaOCl dosing pump was set adjusting a concentration of 6.9 mg/L dosed free Cl_2_. The H_2_O_2_ concentration (quenching agent) was around 3.2 mg/L. The UV sensor signal was 224 ± 21 W/m^2^.

#### 2.6.4. Exp. B: Variation of WWTE Dilutions with Spiked Tap Water

**Figure 4:**
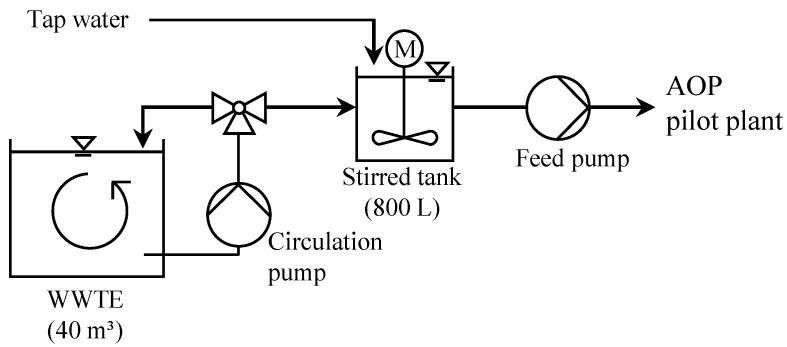
Scheme of the process setup in UV/chlorine AOP Exp. B with different WWTE dilutions.

40 m^3^ WWTE were collected in a tank in the micro sieves hall (Figure 4). With this WWTE, three 800 L batches with different tap water (TW) to WWTE ratios were prepared (530 L TW and 270 L WWTE, 270 L TW and 530 L WWTE, 800 L WWTE) and treated separately as described as follows (the batch regarding sole TW was already investigated in Exp. A (0 mg/L NH_4_^+^-N)). At first, the stirred tank was filled with WWTE and then with tap water. At the same time, the carbamazepine and diclofenac stock solutions were added to the tank. In order to achieve a good homogenization, the tank was stirred for 1 h. For each of the three batches, the general procedure described in Section 2.6.2 was performed separately (duplicate samples taken for the analysis). For all batches, the flow rate of the NaOCl dosing pump was set adjusting a concentration of 6.9 mg/L dosed free Cl_2_. The H_2_O_2_ concentration (quenching agent) was around 3.2 mg/L. The UV sensor signals were 243 W/m^2^ (800 L TW), 175 ± 11 W/m^2^ (530 L TW and 270 L WWTE), 128 ± 8 W/m^2^ (270 L TW and 530 L WWTE), 101 ± 8 W/m^2^ (800 L WWTE).

#### 2.6.5. Exp. C: Variation of CC Concentration on WWTE

In this experiment, NH_4_^+^-loaded WWTE was withdrawn from the micro sieves effluent directly. At first, the general procedure was performed as described in Section 2.6.2, but with the UV lamp switched off. Due to very high NH_4_^+^ concentrations in the WWTE (Table 1), free Cl_2_ from the dosed NaOCl solution reacted immediately to form CC, so no FAC could be detected in the UV chamber influent. Thus, a 1 mg/L total Cl_2_ concentration (1 mg/L CC, 0.0 kWh/m^3^) in the UV chamber influent was adjusted. Now, the UV lamp was switched on and set to 0.4 kW (75 ± 5 W/m^2^). After 10 min, further treated samples were collected. Now, the quenching agent H_2_O_2_ dosage via the second dosing pump was switched on (3.0–4.5 mg/L). After 10 min, the next treated samples were taken from the upper sampling tap. This procedure was repeated for 3 mg/L CC and 5 mg/L CC (both with and without 0.4 kW UV power, both with and without quenching agent dosage) on two different days. Each time triplicate samples were taken for the analysis.

#### 2.6.6. Exp. D: Variation of Flow Rate

In this experiment, WWTE was withdrawn directly from the micro sieves effluent. Through the whole experiment, a FAC concentration of 3 mg/L in the UV chamber influent was set. At first, the general procedure was performed as described in Section 2.6.2 (0.4 kW, 1 m^3^/h, 0.40 kWh/m^3^, 106 W/m^2^). Next, the flow rate was increased to 2 m^3^/h, subsequently repeating the general procedure (0.4 kW, 0.20 kWh/m^3^, 98 W/m^2^, no reference sample taken). The same procedure was repeated with a flow rate of 3 m^3^/h (0.4 kW, 0.13 kWh/m^3^, 92 W/m^2^). Each time triplicate samples were taken for the analysis. The concentration of quenching agent was 3.8–5.8 mg/L H_2_O_2_.

### 2.7. Analytical Methods

#### 2.7.1. Free Cl_2_ (FAC, RFC), Combined Cl_2_ (CC), Total Cl_2_

For the on-site determination of free Cl_2_ and total Cl_2_ equivalent concentrations, a DPD powder pillow method was used (Hach, photometer SQ 118, Merck) (DPD: *N*,*N*-diethyl-*p*-phenylenediamine). The concentration of dosed Cl_2_ was calculated from the flow rates of the feed pump, the dosing pump and the NaOCl stock solution concentration [7]. With ‘other Cl-containing reaction products’ (OCRP) the difference between dosed Cl_2_ and measured total Cl_2_ is described (e.g., OCRP can be chloride). During all experiments, Cl_2_ measurements were carried out as soon as a certain state of equilibrium was achieved.

#### 2.7.2. Emerging Contaminants

Each 1 liter sample was pretreated with 15 mg of the reducing agent sodium thiosulfate (Na_2_S_2_O_3_). The determination of ECs was performed via gas chromatography directly coupled with a mass selective spectrometer (5890N Series II GC, Hewlett Packard, Palo Alto, CA, United States, Hewlett Packard 5972 Series detector, column: VF-Xms, length: 30 m, diameter: 0.25 mm, film thickness: 0.25 µm, Varian, Palo Alto, CA, United States). After the addition of internal standards, the samples were liquid-liquid extracted (dichloromethane, 2 × 40 mL) and evaporated to 100 µL. Quantification was done using the isotope dilution method and external calibration. The limit of quantification (LOQ) was 1 ng/L.

#### 2.7.3. Other Parameters

The temperature and pH value were measured on-site in measuring cells of the pilot plant using a single junction combination electrode sensor by Wallace & Tiernan. The electrical conductivity was measured by means of a WTW TetraCon 325 conductivity detector and a WTW Multi 350i device. The NH_4_^+^-N concentration (Hach LCK 304) and COD (Hach LCK 414, 5 mg/L LOQ) were determined with cuvette rapid tests without prior treatment. The COD cuvettes were heated in a thermostat (Hach HT 200S) for 2 h at 148 °C. The DOC concentrations (1.5 mg/L LOQ) were measured by means of the thermo-catalytic UV oxidation method implemented in the multiN/C 3000 device (Analytik Jena, Jena, Germany). Prior to this analysis, each sample was acidified by hydrochloric acid (pH 2) and filtered (cellulose nitrate, 0.45 µm pore size).

#### 2.7.4. Number of Measurements

The given values in diagrams or tables are mean values calculated from two or three equivalent samples taken consecutively (see experiment descriptions). Error bars in diagrams as well as numbers after the ±  symbol in tables correspond to the calculated standard deviation.

## 3. Results and Discussion

### 3.1. Chlorine Species

#### 3.1.1. Exp. A: Variation of NH_4_^+^-N Concentration in Spiked Tap Water

**Figure 5:**
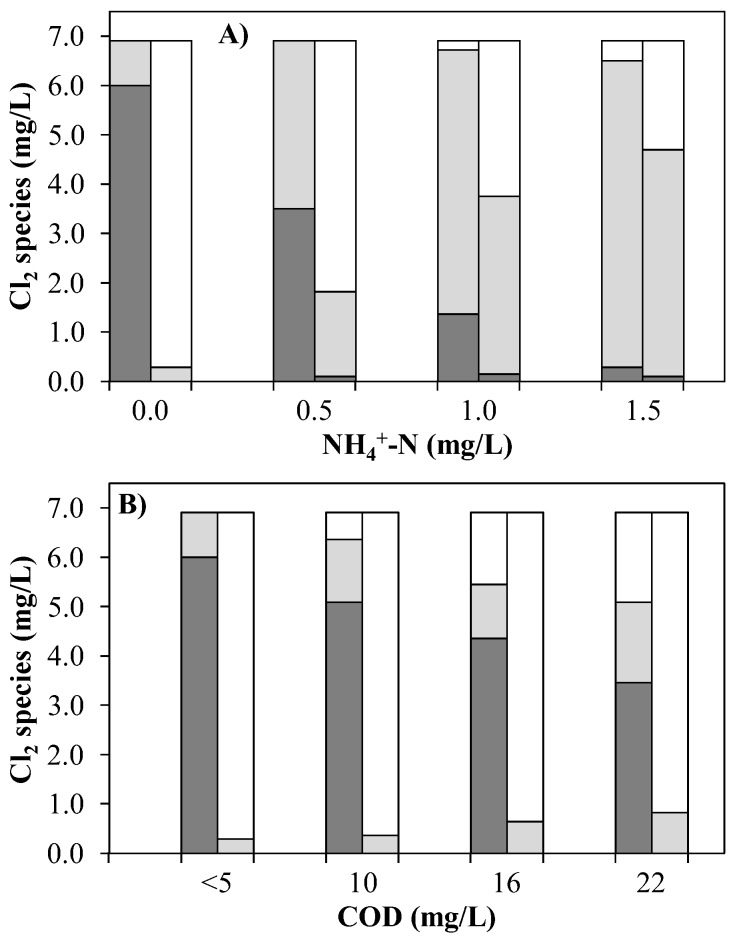
FAC/RFC (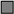), CC (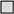) and OCRP (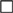) in the UV chamber influent (left columns) and after UV treatment at 0.4 kW and subsequent quenching with H_2_O_2_ (right columns) by means of an AOP pilot plant. (**A**) Exp. A (1 m^3^/h TW, mainly <5 mg/L COD) and (**B**) Exp. B (1 m^3^/h, dilutions of WWTE, <0.1 mg/L NH_4_^+^-N).

In Figure 5, the left columns depict the measured concentrations of FAC, CC and OCRP in the UV chamber influent of Exp. A and B. The right columns show the concentrations of these chlorine species in the pilot plant effluent after quenching with H_2_O_2_.

With RFC concentrations <0.1 mg/L in the pilot plant effluent, it is evident from all experiments that by quenching with H_2_O_2_ a good removal of that proportion of chlorine that had not reacted in the UV reactor (not determined) was achieved. As a rule, this required a concentration of around 3.2 mg/L H_2_O_2_, which corresponds to 95 µM. For the complete reaction of free Cl_2_, the stoichiometric equivalent of H_2_O_2_ is theoretically sufficient [19], which corresponds to 6.7 mg/L Cl_2_. Thus, since the maximum concentration in the pilot plant influent was only 6 mg/L Cl_2_, the H_2_O_2_ dosage of 3.2 mg/L H_2_O_2_ was theoretically sufficient. The reaction rate for this case (*k*_HO__2_^−^_–HOCl_ = 4.4 × 10^7^ M^−1^s^−1^ [19]) is so high that the quenching can take place within a few milliseconds. A contact time of about 4–6 s between the H_2_O_2_ dosing point and the pilot plant effluent was therefore more than sufficient.

Although Exp. A was carried out with tap water, which in traces had only been spiked with carbamazepine and diclofenac, and the DOC of which was below the LOQ of 1.5 mg/L, in the case of no ammonium chloride spiking, a dosage of 6.9 mg/L Cl_2_ was required to obtain a concentration of 6 mg/L FAC in the UV chamber influent (Figure 5A). The slightly higher dosage was due to hardly noticeable organic impurities that were present in the tap water or e.g., residual impurities in the stirring tank, the pump hoses or in the static mixers (the plant was thoroughly flushed before each experiment, however, a 100 percent cleaning was challenging). For all NH_4_^+^ concentrations examined, the same dosage concentration of 6.9 mg/L Cl_2_ was used. As expected, the increase in ammonium ion concentration led to a decrease of the FAC concentration in the UV chamber influent. At all NH_4_^+^ concentrations studied, furthermore, lost free chlorine was found almost entirely in the form of combined chlorine, i.e., in the form of chloramines. Thus, a concentration of 1 mg/L NH_4_^+^-N (71 µM) already reduced the achievable FAC concentration by 75%. The associated loss of 4.7 mg/L free Cl_2_ (66 µM) was quasi-equimolar with the NH_4_^+^-N concentration of 71 µM. Accordingly, a stoichiometric inhibition of the UV/chlorine AOP is to be expected in tap water by ammonium ions (inhibition ratio of 4.7 mg FAC per mg NH_4_^+^-N between 0 and 1 mg/L NH_4_^+^-N) (Figure S1).

Margerum et al. [20] as well as Qiang and Adams [21] found an apparent rate constant of 1.3 × 10^4^ M^−1^s^−1^ for the reaction of HOCl with NH_4_^+^ at 25 °C and pH 7 [22]. Using this value, a contact time of 5.6 s between the dosage point of chlorine and the Cl_2_ measuring cell could be determined with the least squares method (Table S2 and Figure S2). Accordingly, on the basis of the diameter of the pipes, a contact time of 6.1 s between the chlorine dosage point and the UV chamber influent was calculated (Table S3). The difference in contact time was therefore only slight, so that the FAC concentration between the UV chamber influent and the measuring cell differed only by a maximum of 0.05 mg/L (Table S2).

Furthermore, it can be seen from Figure 5A that the CC concentration in the pilot plant effluent was always lower than in the UV chamber influent for all tested batches (with an increase in NH_4_^+^ concentration from left to right, the degree of CC elimination changed as follows: 68, 50, 33, 26%). A specific proportion of the chloramines present in the UV chamber influent was therefore degraded in the UV chamber. The elimination of CC by UV light is a familiar phenomenon. Yang et al. [9] found a similar decrease from 2.1 to 1.6 mg/L monochloramine in ammonium-rich wastewater (pH 7) by UV light (10 W). Chuang et al. [23] also found that at pH 7 NH_2_Cl is reduced up to 50% at fluences of up to 3000 mJ cm^−2^. The weakly pronounced falsification of the result of the CC concentration in the pilot plant effluent by the quenching agent H_2_O_2_ as quantified as 0.0388 mg total Cl_2_/mg H_2_O_2_ [7] is estimated to be very low.

#### 3.1.2. Exp. B: Variation of WWTE Dilutions with Spiked Tap Water

Since the COD is an adequate parameter to describe the cumulative organic load of WWTE, all dilutions in Exp. B are classified by their initial COD (Figure 5B). In Table 1, the exact measured COD values of the dilutions can be seen. For simplification reasons, these COD values were simplified to 10, 16 and 22 mg/L COD in Figure 5B. The COD of pure tap water could therefore be calculated to approx. 4 mg/L (<5 mg/L). In all batches, NH_4_^+^-N was always <0.1 mg/L, which allowed the investigation of the sole influence of COD, i.e., organic and some inorganic compounds in WWTE, on the UV/chlorine process. As in Exp. A, for all of the four batches in Exp. B, always the same NaOCl stock solution dosage of 6.9 mg/L Cl_2_ was applied. The FAC concentration obtained in the UV chamber influent decreased linearly proportional to the COD up to 22 mg/L COD at a ratio of 0.16 mg FAC per mg COD (Figure S3). As already reported by Rott et al. [7] in the case of undiluted effluent from a WWTP, therefore, to obtain the desired FAC concentration in the UV chamber influent approximately the double dosage was necessary.

CC increased slightly with an increase in COD (approx. 0.03 mg CC/mg COD). In the UV chamber, however, CC was eliminated between 40 and 70%. Compared to Exp. A, a far greater proportion of OCRP was found in the UV chamber influent, which also increased at a significantly higher ratio of 0.1 mg OCRP/mg COD. This is obvious, as in Exp. B chlorine reacted predominantly with organic compounds, not all chlorinated products of which can necessarily be detected as CC using the DPD method [7].

Knowing the exact contact time of 5.6 s between the dosing point of chlorine and the Cl_2_ measuring cell from Exp. A and the recorded FAC concentrations in this measuring cell at known COD concentrations, the least squares method could be used to determine the rate constant between HOCl and COD to be 182 M^−1^s^−1^ (the COD is not the actual reaction partner of HOCl, but represents the sum of all organic compounds in the sample, Table S4 and Figure S4). The COD of the investigated WWTE of 22 mg/L was typical for the investigated WWTP and thus representative, albeit slightly above the annual mean value of 19.7 mg/L (Figure 1). Since COD limit values usually depend on the size class of WWTPs and can even be in the three-digit mg/L range, it should be considered that such WWTPs would require relatively high Cl_2_ doses. Assuming the abovementioned rate constant of HOCl with COD, for instance, at a COD of 80 mg/L in WWTE (neglecting NH_4_^+^) about 34 mg/L of dosed Cl_2_ would be required to obtain a desired FAC concentration of 3 mg/L in the UV chamber influent (Table S5).

Whether ammonium ions or organic pollution play a major role in FAC inhibition during the entire operating year of a WWTP, is very case-specific. The following calculation intends to solve this question for the year of operation of the LFKW shown in Figure 1. In this year, the annual mean value of the NH_4_^+^-N concentration in WWTE was 1.56 mg/L and the COD average was 19.7 mg/L. Based on the abovementioned inhibition ratios, because of ammonium ions the required Cl_2_ dosage to obtain 3 mg/L FAC on average in the UV chamber influent would have been 10.3 mg/L, with 7.3 mg/L of it being inhibited by ammonium ions on average. Due to the organic constituents (COD), the required average Cl_2_ dosage to obtain 3 mg/L FAC in the UV chamber influent would have been 6.2 mg/L, with 3.2 mg/L FAC of it being inhibited on average (under the simplified assumption that the required Cl_2_ dosage to obtain a specific FAC concentration is linearly proportional to the COD at ≤30 mg/L COD (Figure S5)—over 95% of the year, this concentration range prevailed in the WWTE). Thus, under very simplified assumptions, this would have resulted in a required annual average Cl_2_ dosage concentration of at least 13.5 mg/L. This shows that on average ammonium ions in the WWTE would have inhibited dosed chlorine more strongly than organic components.

NH_4_^+^-N concentrations of 5–10 mg/L, for example, would result in minimum dosages of 27–54 mg/L Cl_2_ to obtain a FAC concentration of 3 mg/L in the UV chamber influent (Table S5). However, such high dosing quantities are highly questionable with regard to the formation of critical by-products. For periods in which such high ammonium ion concentrations prevail, it would therefore be decisive for the applicability of the UV/chlorine AOP whether CC also causes a sufficiently efficient EC elimination due to its activation with UV light.

#### 3.1.3. Exp. C: Variation of CC Concentration on WWTE

**Figure 6:**
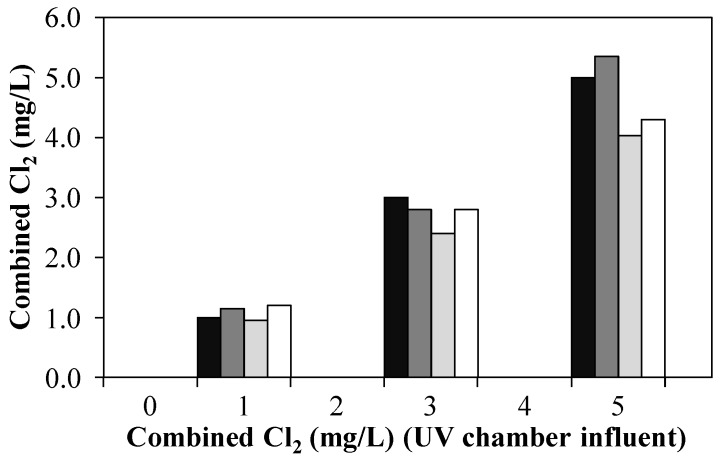
Combined Cl_2_ in the UV chamber influent (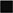) and pilot plant effluent after treatment of 1 m^3^/h NH_4_^+^-loaded WWTE with CC (no UV) (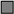), UV/CC (0.4 kW, no quenching) (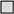) and UV/CC (0.4 kW, 3.0–4.5 mg/L H_2_O_2_ quenching) (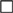) by means of an AOP pilot plant (Exp. C).

In the experiment investigating the efficiency of the UV/CC AOP, a sufficiently high NH_4_^+^ concentration was present in the WWTE at all three CC concentrations tested (1, 3, 5 mg/L). Consequently, the dosed free Cl_2_ reacted quickly to form CC and was thus almost completely detected in the UV chamber influent as CC (OCRP concentrations in the UV chamber influent at 1, 3, 5 mg/L CC were: 0.6 mg/L, 0.2 mg/L, 0.1 mg/L (not shown in Figure 6)). The COD of the raw samples varied only slightly between 24 and 31 mg/L. When the UV lamp was off, the CC concentration between the UV chamber influent and the pilot plant effluent did not change significantly (Figure 6). Only when the UV lamp was on (operated at 0.4 kW), the CC concentration dropped between 5 and 20%, indicating activation/decay of chloramines possibly according to Equation (5).

Within the scope of this work, it was not investigated which compounds exactly made up the CC. However, since the rate constant of chlorine with ammonium ions is almost one hundred times greater than the rate constant of chlorine with COD (see Section 3.1.1 and Section 3.1.2), it can be assumed that mainly inorganic chloramines were formed in the presence of ammonium ions. When H_2_O_2_ quenching was carried out additionally, the CC concentrations found in the pilot plant effluent were slightly higher than those without quenching. This is because H_2_O_2_ leads to a slight falsification of the total Cl_2_ determination method with DPD [7,24]. However, this falsification is not significant enough to interfere with the conclusion that CC cannot be quenched with H_2_O_2_. Assuming that most of the CC was composed of inorganic chloramines, this is obvious since the rate constants of monochloramine (*k*_NH__2__Cl–H__2__O__2_ = 2.76 × 10^−2^ M^−1^s^−1^ [25]) and dichloramine (*k*_NHCl__2__–H__2__O__2_ = 3.60 × 10^−6^ M^−1^s^−1^ [25]) with H_2_O_2_ are very low.

Chlorinated compounds are considered environmentally critical and should not be simply discharged with the WWTE into the receiving water. The fact that CC was only slightly eliminated when the UV lamp was on and CC could not be sufficiently removed by quenching with H_2_O_2_ shows that high NH_4_^+^ concentrations in the WWTE make the UV/CC AOP seem impractical at the investigated UV power range.

#### 3.1.4. Exp. D: Variation of Flow Rate

**Figure 7:**
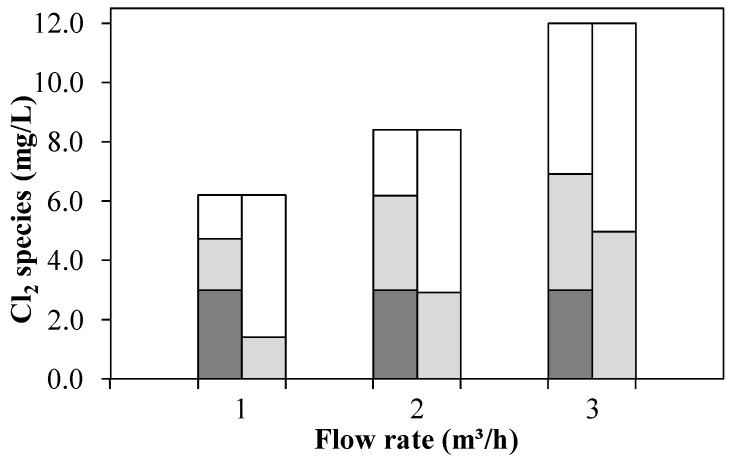
FAC/RFC (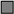), CC (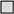) and OCRP (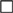) in the UV chamber influent (left columns) and after UV treatment and subsequent quenching with H_2_O_2_ (right columns) by means of an AOP pilot plant at 0.4 kW (Exp. D: WWTE, 0.13–0.57 mg/L NH_4_^+^-N).

From Figure 7 it becomes apparent that Exp. D was carried out at a time when the NH_4_^+^-N concentration slowly increased from 0.13 to 0.57 mg/L during the experiment. Thus, at a flow rate of 1 m^3^/h, approximately twice the Cl_2_ dosage amount was required to obtain 3 mg/L FAC in the UV chamber influent, whereas at 3 m^3^/h this was only the case at four times the amount. This was accompanied by an increasing CC and OCRP concentration with increasing flow rate. The CC concentration was hardly reduced by the UV irradiation, at 3 m^3^/h it even increased slightly. The lack of CC elimination at higher flow rates indicates that at 3 m^3^/h the wastewater passed the UV chamber too quickly (2–3 s) resulting in no sufficient time for CC photolysis. The slight increase can be attributed to a slight falsification of the DPD method by H_2_O_2_.

### 3.2. Emerging Contaminants

#### 3.2.1. Exp. A: Variation of NH_4_^+^-N Concentration and Exp. B: Variation of WWTE Dilutions

**Figure 8:**
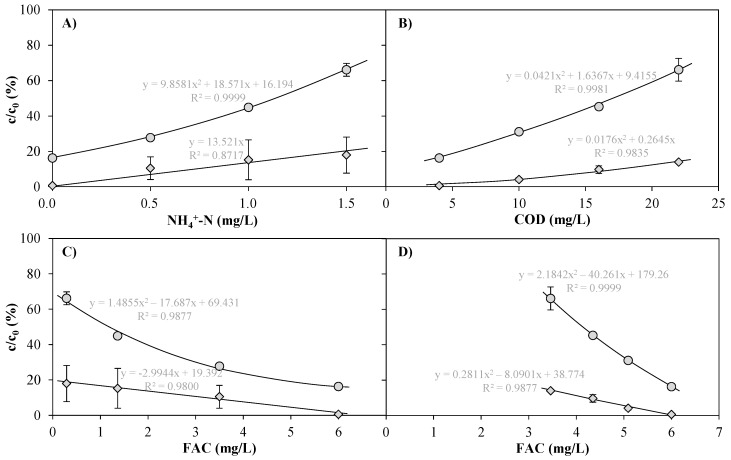
Carbamazepine (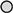) and diclofenac (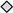) after treatment of tap water spiked with NH_4_Cl (Exp. A, <5 mg/L COD) and dilutions of WWTE with tap water (Exp. B, <0.1 mg/L NH_4_^+^-N) by means of an AOP pilot plant at 1 m^3^/h, 6.9 mg/L dosed Cl_2_, 0.4 kW UV lamp power (0.4 kWh/m^3^ electrical energy consumption) and subsequent quenching with around 3.2 mg/L H_2_O_2_ as functions of NH_4_^+^-N (**A**); COD (**B**) and FAC concentration as detected in the UV chamber influent (**C**: Exp. A; **D**: Exp. B). For E_EO_ values, see Table S6.

Figure 8A shows the residual concentrations of carbamazepine (CBZ) and diclofenac (DCF) as a function of the ammonium ion concentration in tap water matrix treated with 6.9 mg/L dosed Cl_2_ at 0.4 kW UV power. Figure 8B shows the dependence of the residual concentrations on different dilutions of WWTE (22 mg/L COD) with tap water (approx. 4 mg/L COD). The elimination of CBZ from tap water without ammonium ions was approx. 84%, whereas DCF was eliminated in this matrix at approx. 99.4%. An increase in NH_4_^+^-N to 1.5 mg/L resulted in a similar deterioration of the degree of elimination for both ECs as an increase in the COD concentration to around 22 mg/L. Accordingly, with the highest NH_4_^+^-N concentration and COD tested, the CBZ elimination was only 33–34% and the DCF elimination was 82–86%. The trend lines could be represented well predominantly by means of square equations.

DCF is very susceptible to photolysis [26]. In experiments with the same UV pilot plant as in this study [7], the UV photolysis at 0.4 kWh/m^3^ in WWTE matrix resulted in 81–90% elimination of this compound, whereas with 3 mg/L FAC in the UV chamber influent (7.3 mg/L dosed Cl_2_) the elimination was only 52–53% (without UV). CBZ, on the other hand, was not eliminated at all with sole FAC treatment, but was removed by 18–22% with UV light only at 0.4 kWh/m^3^ electrical energy consumption (no chlorine dosage) in WWTE matrix. It can therefore be assumed that the degradation of the latter compound as found in Figure 8 was mainly caused by radicals, whereas for DCF UV light was sufficient to degrade the molecule, and chlorine radicals but also free chlorine only slightly contributed to an improved elimination.

Soufan et al. [27] observed a third-order reaction between CBZ and HOCl at pH 7 (145 M^−2^s^−1^), whereas the reaction between DCF and HOCl was found to be of second-order (3.5 M^−1^s^−1^) [28]. These kinetic rate constants were determined at initial EC concentrations of 10 µM (3 mg/L DCF, 2.4 mg/L CBZ). The investigated [HOCl]/[CBZ] ratio was between 37 and 550, whereas the one of [HOCl]/[DCF] was 17–33. In this work, however, the EC concentrations were so low that the [HOCl]/[EC] ratio was between 10,000 and 25,000. Accordingly, it is not surprising that DCF half-lives of more than 30 min (and DCF is the more reactive EC), as calculated by using the rate constant 3.5 M^−1^s^−1^, were not applicable to the study presented here. For the same reason, the comparatively low rate constant of *k_obs_* = 0.78 min^−1^ for CBZ degradation by UV/chlorine (2 mg/L CBZ (8.5 µM), 280 µM Cl_2_, 1.48 mW/cm^2^ (41 W), pH 7, in pure water) as found by Wang et al. [15] was not transferable to the results of this study as well.

Figure 8C,D clearly show that the similar degrees of elimination of the two ECs between Exp. A and Exp. B did not correlate with the FAC concentration in the UV chamber influent. This indicates that the elimination of ECs cannot be traced back to FAC alone. The CC concentrations in the UV chamber influent resulting from the different NH_4_^+^-N concentrations were considerably higher (0.9–6.2 mg/L CC) than those resulting from the different COD concentrations (0.9–1.6 mg/L CC). On the other hand, the FAC concentrations were considerably lower. Despite these lower FAC concentrations in the presence of NH_4_^+^, the elimination of ECs was similar in the investigated measuring range with both ammonium ions and COD. It is obvious that in Exp. A CC was composed of inorganic chloramines, which decompose to radicals by UV light (Equation (5) [6,9]). Figure 5A also shows that CC was degraded partially in the presence of UV irradiation. It can therefore be assumed that this conversion of chloramines into radicals by UV light also contributed to the degradation of the ECs.

#### 3.2.2. Exp. C: Variation of CC Concentration on WWTE

**Figure 9:**
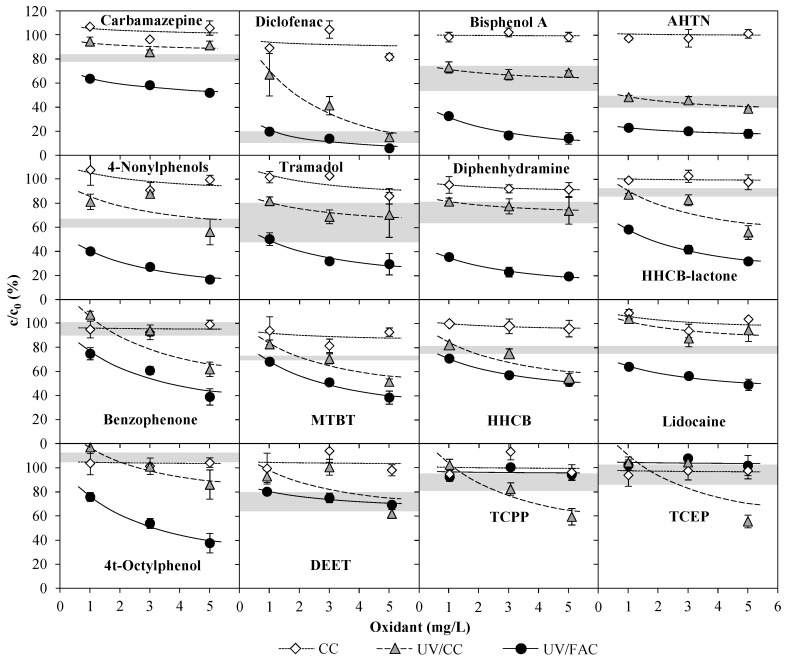
Emerging contaminants found in WWTE after treatment with the UV/FAC AOP [7], the UV/CC AOP (Exp. C) at 0.4 kWh/m^3^ electrical energy consumption (0.4 kW) and with CC alone (no UV, Exp. C) as a function of the oxidant concentration (1 m^3^/h flow rate). Gray line: sole UV treatment at 0.4 kWh/m^3^ (1 m^3^/h flow rate) [7]. For E_EO_ values, see Table S7.

In Table 1 and Table S1, the initial concentrations of the ECs in each reference sample of Exp. C can be seen. Thus, for the vast majority of ECs analyzed, the initial concentrations did not differ significantly. In Figure 9, the results of Exp. C (sole CC and UV/CC treatment) are compared to those obtained in an experiment with effluent of the same WWTP with varied FAC concentrations in the UV chamber influent of the same UV pilot plant at 0.4 kWh/m^3^ (UV/FAC) [7]. Furthermore, a solid gray line demonstrates the residual EC concentrations after sole UV treatment (0.4 kWh/m^3^) (range of standard deviation taken from Rott et al. [7]).

When no UV radiation was applied and only CC was present in the UV chamber influent, there was no elimination of the ECs in the WWTE. It is evident that the dosed Cl_2_ reacted quickly with the ammonium ions in the wastewater (*k* = 1.3 × 10^4^ M^−1^s^−1^ [20,21]). These ammonium ions thus competed with the ECs for free Cl_2_ [29]. Chloramines (it can be assumed that the majority of CC consisted of inorganic chloramines, see Section 3.1.3) can also react with ECs, but this reaction is significantly slower than with free Cl_2_ [29]. This clearly shows that the oxidizing ability of inorganic chloramines is not sufficient for compounds that are present in traces to be significantly degraded within the very short contact time of less than 30 s prevailing in the pilot plant.

In order to be able to assess the actual elimination effect of the AOPs, knowledge of the elimination performance of the 16 ECs by treatment with Cl_2_ alone is required. In an experiment with 3 mg/L FAC at 1 m^3^/h [7], only the following compounds were eliminated with sole FAC treatment: 4t-octylphenol (44% residual concentration), MTBT (64%), tramadol (64%), DCF (47%), diphenhydramine (35%), bisphenol A (28%) and 4-nonylphenols (20%). All other compounds (including CBZ) were not significantly degraded by 3 mg/L FAC. Thus, particularly the elimination of ECs such as CBZ, AHTN, HHCB, HHCB-lactone, benzophenone and lidocaine, which were not eliminated significantly by Cl_2_ alone and the degree of elimination of which differed markedly between sole UV and UV/chlorine treatment, can be traced back to reaction with radicals.

For all compounds, the elimination effect of 1–3 mg/L CC with simultaneous UV treatment (UV/CC) was not significantly higher than the elimination effect by sole UV treatment. The fact that ammonium ions were present in the pilot plant influent thus had a decreasing effect on the EC elimination performance. Accordingly, due to the rapid reaction of free Cl_2_ with ammonium ions, the •OH radical yield was considerably reduced [30]. Furthermore, the oxidizing ability of chloramines is significantly lower than that of HOCl [30]. In addition, radicals can also be consumed for the oxidation of ammonium ions to nitrite and nitrate ions [6,17]. On the other hand, HHCB, HHCB-lactone, benzophenone, MTBT, TCEP and TCPP seemed to be affected by a CC concentration of 5 mg/L at UV/CC (still, UV/CC was less effective than UV/FAC except for TCEP, TCPP). Especially for the latter ECs, however, it is questionable why UV/CC worked better than UV/FAC, although HOCl (Φ_254 nm_ = 1.5, Φ_200–350 nm_ = 3.3–4.0) has a significantly higher quantum yield than monochloramine (Φ_254 nm_ = 0.3, Φ_200–350 nm_ = 0.7) [6]. During the experiment, TCEP and TCPP were the ECs with the strongest variation in initial concentration (e.g., 0.37 µg/L TCEP at 3 and 5 mg/L CC and 2.19 µg/L TCEP at 1 mg/L CC). In contrast, the initial concentrations of the other ECs were usually on a similar scale. Thus, the supposedly better elimination can possibly be traced back to the changing wastewater composition and considerably different initial concentration during the course of the experiment. The very fact that the ammonium ion concentration in the WWTE varied considerably within a few minutes indicates that the nitrification of the WWTP did not function optimally at this moment. A non-functioning nitrification can also indicate a non-functioning elimination of other organic or inorganic compounds and thus a very different wastewater matrix as compared to the regular operation. Based on the few examples, a better efficiency of UV/CC compared to UV/FAC should therefore not be concluded for the organophosphoric acid esters without further research.

The effect of the changing wastewater composition could also be observed very well with DCF, 4-nonylphenols, lidocaine and DEET. Here, in parts the UV/CC combination was even less effective than sole UV treatment. The question now arises as to whether the changing wastewater composition during the experiment had a negative effect on the reliability of the results. It must be noted that high ammonium ion concentrations in WWTE are the exception in WWTP operation. Since nitrification obviously does not function reliably when the ammonium ion concentration is elevated, the ammonium ion concentration changes steadily, i.e., an equilibrium state of WWTE cannot be established for such experiments. Furthermore, a changing NH_4_^+^ concentration also indicates a poorer elimination of other compounds in the wastewater (e.g., occurring solids or other nitrogenous compounds may react with chlorine; color change of the wastewater may lead to a stronger absorption of UV light), so that other conditions may prevail for the pilot plant. This experiment should cover this exceptional case and is therefore representative.

In the experiments by Yang et al. [9], the elimination of some pharmaceuticals spiked in ammonium-rich wastewater (3.14 mg/L NH_4_^+^-N) was investigated. For example, at a dosage of 5 mg/L Cl_2_, 10 W UV power, pH 7 and a contact time of 1.5 min, with 30% the elimination of CBZ was significantly lower than in wastewater with less than 0.03 mg/L NH_4_^+^-N. However, the degradation could be mainly attributed to chlorine radicals, which disagreed with the findings of this work for CBZ (no significant difference in elimination between UV and UV/CC). The comparison of both results demonstrates that the contact time in the UV chamber of 6–10 s is not sufficient for EC elimination when instead of FAC only CC is present in the UV chamber influent.

Compared to UV, CC, and UV/CC treatment, the UV/FAC process was the most effective method. Here, for many compounds (e.g., CBZ, HHCB-lactone, HHCB, benzophenone, MTBT) the degree of elimination differed significantly from the degree of elimination by sole UV and sole FAC treatment. It was already worked out by Rott et al. [7] that, in the dosing range of 1–6 mg/L oxidant, the UV/H_2_O_2_ process is significantly less effective than the UV/FAC process in terms of EC elimination. However, it is important to point out that especially in cold seasons due to insufficient nitrification, the ammonium ion concentration in the WWTE can become so high that it becomes impossible to achieve sufficiently high FAC concentrations in the UV chamber influent. In such cases, the UV/chlorine AOP becomes the UV/CC AOP. The fact that the degrees of elimination during UV/CC treatment differed insignificantly from sole UV treatment clearly indicates that the UV/chlorine AOP is very sensitive and loses its effectiveness drastically as soon as there is no FAC but only CC in the UV chamber influent. At lower ammonium ion concentrations, this may be compensated by an increased dosage of Cl_2_. Considering by-product formation, however, at very high ammonium ion concentrations it is questionable whether the required Cl_2_ dosage goes hand in hand with the concept of environmentally friendly wastewater treatment. Furthermore, it was shown that CC cannot be quenched with H_2_O_2_ (Section 3.1.3). The increased by-product emission can therefore not be met with H_2_O_2_ quenching.

#### 3.2.3. Exp. D: Variation of Flow Rate

**Figure 10:**
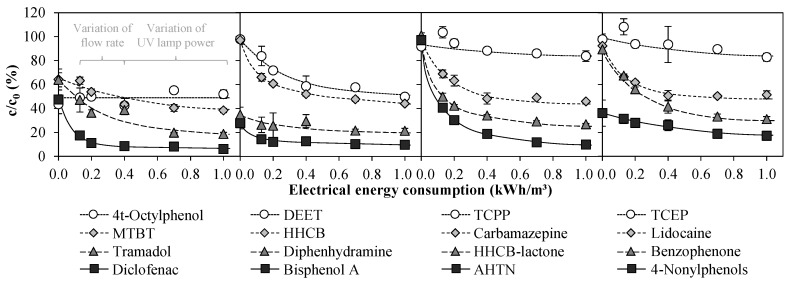
Emerging contaminants after treatment of WWTE by means of an AOP pilot plant as a function of the electrical energy consumption at 3 mg/L FAC in the UV chamber influent and subsequent quenching with H_2_O_2_. The results of two experiments are shown: variation of flow rate between 1 and 3 m^3^/h at 0.4 kW UV lamp power (Exp. D) and variation of UV lamp power between 0 and 1 kW at a flow rate of 1 m^3^/h (taken from Rott et al. [7]). For E_EO_ values, see Table S8.

Figure 10 summarizes the residual concentrations of ECs in WWTE from two different experiments. 

In an experiment by Rott et al. [7] with the same pilot plant applying a flow rate of 1 m^3^/h and 3 mg/L FAC in the UV chamber influent, the UV power was varied between 0 and 1 kW. In Exp. D of this study, both the UV power and the FAC concentration in the UV chamber influent were kept constant at 0.4 kW and 3 mg/L, whereas the flow rate of the pilot plant was varied between 1 and 3 m^3^/h. The results of both experiments are shown as a function of the electrical energy consumption.

For many of the ECs investigated, it can be seen very well that the degree of elimination was negatively influenced by higher flow rates, i.e., lower electrical energy consumption. This is obvious, since doubling or tripling the flow rate is accompanied by shortening the contact time in the UV reactor by half and to one third, respectively. Increased UV power at a constant flow rate, i.e., higher electrical energy consumption, had a positive effect on the degree of EC elimination. Thus, applied over the electrical energy consumption, the results of two relatively different experiments proved to fit well together.

Compared to sole FAC dosage of 3 mg/L (0 kWh/m^3^), a significant improvement in the elimination of several ECs was achieved by addition of UV light. The ECs AHTN, HHCB-lactone, benzophenone, HHCB and CBZ were best eliminated with >50 percentage points (p.p.) difference between sole Cl_2_ and UV/Cl_2_ treatment. 40–50 p.p. differences were achieved for DEET, tramadol, lidocaine and DCF. Between 0.13 and 1.00 kWh/m^3^, the differences in the elimination of most ECs were small. Within this range, the variation of electrical energy consumption had the greatest effect on the ECs benzophenone, DEET and AHTN with more than 30 p.p. difference between the minimum and maximum degree of elimination. The smallest influence with less than 12 p.p. difference was found for diphenhydramine, DCF, and bisphenol A. However, this number is so low because even at very low electrical energy consumption high degrees of elimination already prevailed and a large leap to 100% elimination was therefore not possible.

The question remains whether the slightly increased CC concentration of up to 3.9 mg/L due to the increase of NH_4_^+^-N concentration in WWTE up to 0.57 mg/L (Table 1) towards the end of Exp. D influenced the EC elimination yields. Although in Exp. A for tap water matrix it was shown that chloramines may contribute to a greater EC elimination, in Exp. C it was observed for WWTE matrix with almost all ECs that CC in combination with UV light did not contribute significantly to a greater EC elimination.

## 4. Conclusions

Many influencing factors such as increased ammonium ion concentrations or increased COD values in WWTE have a negative effect on the maintenance of FAC in the UV chamber influent, which, as experiments of this work showed, is essential for the UV/chlorine AOP to effectively eliminate ECs. Within the electrical energy consumption range tested (0.13–1 kWh/m^3^), a stable EC elimination performance of the UV/chlorine AOP can therefore not be maintained regularly throughout the year. To meet this problem, additional treatment steps would be a way of maintaining a good elimination performance. One possibility is to operate the UV AOP system with NaOCl only in the case of low ammonium ion concentrations and to dose H_2_O_2_ (UV/H_2_O_2_ AOP) instead of NaOCl at elevated ammonium ion concentrations, although in this case the EC elimination yields may decrease significantly [7]. In any case, the installation of an activated carbon stage downstream of the UV/chlorine AOP is recommended due to significant formation of adsorbable organohalogens (AOX) in the UV/chlorine AOP [7]. Since the entire process technology strongly depends on the fluctuating wastewater composition, for an immediate reaction to these changes, a very advanced control technology and process engineering would be required. Despite these issues, the UV/chlorine AOP has advantages over the UV/H_2_O_2_ AOP in terms of EC removal, hygienization and total estrogenic activity elimination [7]. Hence, further research at pilot scale would have to investigate whether shorter contact times between the chlorine dosing point and the UV chamber or higher UV lamp powers might contribute to weaker by-product formation and a more stable EC elimination performance.

## Figures and Tables

**Table 1:** Initial parameter values c_0_ measured in the reference samples.

Experiment		T	pH	COD	DOC	NH_4_^+^-N	Cond.	CBZ	DCF
		°C	-	mg/L	mg/L	mg/L	µS/cm	µg/L	µg/L
A	<0.1 mg/L NH_4_^+^-N	16.3	7.4	<5.0	<1.5	<0.1	334 ± 0	0.25 ± 0.00	1.70 ± 0.01
	0.5 mg/L NH_4_^+^-N	16.0	8.0	<5.0	<1.5	0.5	341 ± 0	0.94 ± 0.02	1.66 ± 0.18
	1.0 mg/L NH_4_^+^-N	16.7	8.0	<5.0	<1.5	1.0	348 ± 3	0.59 ± 0.03	1.90 ± 0.03
	1.5 mg/L NH_4_^+^-N	15.5	8.0	<5.0	<1.5	1.5	350 ± 0	1.07 ± 0.04	1.40 ± 0.25
B	<5 mg/L COD	16.3	7.4	<5.0	<1.5	<0.1	334 ± 0	0.25 ± 0.00	1.70 ± 0.01
	10 mg/L COD	17.7	8.2	12.1 ± 1.6	3.1 ± 0.2	<0.1	520 ± 1	0.40 ± 0.00	3.08 ± 0.07
	16 mg/L COD	17.1	8.2	16.2 ± 0.1	4.2 ± 0.4	<0.1	717 ± 4	0.48 ± 0.07	3.01 ± 0.37
	22 mg/L COD	18.2	8.2	21.0 ± 1.5	5.1 ± 0.1	<0.1	909 ± 1	0.48 ± 0.01	2.08 ± 0.51
C	1 mg/L CC	13.9	6.9	31.2 ± 1.0	8.3 ± 0.9	1.58 ± 0.02	920 ± 0	0.68 ± 0.05	2.67 ± 0.18
	3 mg/L CC	14.8	7.0	23.0 ± 0.8	5.9 ± 0.3	6.26 ± 0.05	973 ± 0	0.57 ± 0.02	1.98 ± 0.25
	5 mg/L CC	14.2	7.0	24.8 ± 1.5	6.5 ± 0.9	6.73 ± 0.85	1048 ± 9	0.57 ± 0.01	3.87 ± 0.45
D	1 m^3^/h flow rate	18.3	6.9	21.9 ± 0.1	4.5 ± 0.0	0.13 ± 0.00	823 ± 0	0.54 ± 0.02	1.31 ± 0.02
	1 m^3^/h flow rate	18.3	6.9	20.6 ± 2.3	n.m.	0.57 ± 0.00	822 ± 6	0.57 ± 0.02	1.42 ± 0.14

n.m.: not measured, T: temperature, COD: chemical oxygen demand, DOC: dissolved organic carbon, Cond.: electrical conductivity, CBZ: carbamazepine, DCF: diclofenac, FAC: free available chlorine, CC: combined Cl_2_.

## Supplementary Materials

Supplementary calculations, figures and tables are available online at http://www.mdpi.com/1660-4601/15/6/1276/s1. Table S1: Initial concentrations of emerging contaminants in all experiments with WWTE. Table S2: Results of least squares method to determine the contact time between Cl_2_ dosage point and Cl_2_ measuring cell. Table S3: Calculation of contact time between Cl_2_ dosage point and UV chamber influent. Table S4: Results of least squares method to determine the kinetic rate constant *k*_HOCl–COD_. Table S5: Determination of regression curves to determine the required Cl_2_ dosage for 3 mg/L FAC in the UV chamber influent as a function of NH_4_^+^-N or COD. Table S6: Electrical energy consumption per order of compound removal E_EO_ as calculated from the data given in Figure 8. Table S7: Electrical energy consumption per order of compound removal E_EO_ (kWh/m³/order) as calculated from the data given in Figure 9. Table S8: Electrical energy consumption per order of compound removal E_EO_ (kWh/m³/order) as calculated from the data given in Figure 10. Figure S1: Measured FAC concentrations in the Cl_2_ measuring cell as a function of the NH_4_^+^ concentration in the pilot plant influent in tap water matrix. Figure S2: Results of least squares method to determine the contact time between Cl_2_ dosage point and Cl_2_ measuring cell. Approximation to measured data by changing the contact time at a fix second order rate constant: *k*_HOCl,NH__₃_ = 1.3×10^4^ M^–1^s^–1^. Figure S3: Measured FAC concentrations and calculated FAC concentrations (*k* = 182 M^–1^s^–1^) in the Cl_2_ measuring cell as a function of the COD concentration in the pilot plant influent (dilutions of wastewater treatment plant effluent). Figure S4: Results of least squares method to determine the kinetic rate constant *k*_HOCl–COD_. Approximation to measured data by changing the kinetic rate constant at a fix contact time of 5.605 s. Figure S5: Required Cl_2_ dosage to obtain 3 mg/L FAC in the UV chamber influent (6.112 s contact time) in the presence either of ammonium ions or COD (the results may differ when both are present at the same time) calculated either using the kinetic rate constants (*k*_HOCl,NH__₃_ = 1.3×10^4^ M^–1^s^–1^ and *k*_HOCl–COD_ = 182.1 M^–1^s^–1^) or the inhibition ratios (4.7 mg FAC per mg NH_4_^+^-N and 0.16 mg FAC per mg COD).
