# Supplementary material for: Influence of Ammonium Ions, Organic Load and Flow Rate on the UV/Chlorine AOP Applied to Effluent of a Wastewater Treatment Plant at Pilot Scale
Source: Int J Environ Res Public Health. 2018 Jun 16;15(6):1276. doi: 10.3390/ijerph15061276 (PMC6025302; doi:10.3390/ijerph15061276)
Supplement: Supplementary file 1 [file ijerph-15-01276-s001.pdf]

International Journal of
Environmental Research and Public Health

MDPI

Supplementary Material to the Article

Influence of Ammonium Ions, Organic Load and Flow Rate on the UV/Chlorine AOP Applied to Effluent of a Wastewater Treatment Plant at Pilot Scale

Eduard Rott *, Bertram Kuch, Claudia Lange, Philipp Richter and Ralf Minke

Institute for Sanitary Engineering, Water Quality and Solid Waste Management, University of Stuttgart, Bandtäle 2, 70569 Stuttgart, Germany
* Correspondence: eduard.rott@iswa.uni-stuttgart.de; Tel.: +49-711-685-60497

A. Initial EC Concentrations

Table S1. Initial concentrations of emerging contaminants in all experiments with WWTE.

Experiment		Bisphenol A (µg/L)	MTBT (µg/L)	AHTN (µg/L)	DEET (µg/L)	HHCB (µg/L)	HHCB-lactone (µg/L)	4t-Octyl-phenol (µg/L)
C	1 mg/L CC	0.85 ± 0.03	0.27 ± 0.03	0.18 ± 0.02	0.08 ± 0.01	1.18 ± 0.10	1.58 ± 0.04	0.03 ± 0.00
	3 mg/L CC	0.84 ± 0.02	0.14 ± 0.01	0.16 ± 0.01	0.04 ± 0.00	1.09 ± 0.05	1.43 ± 0.10	0.02 ± 0.00
	5 mg/L CC	0.80 ± 0.10	0.27 ± 0.01	0.18 ± 0.01	0.07 ± 0.00	1.09 ± 0.03	1.38 ± 0.05	0.03 ± 0.00
D	1 m³/h flow rate	0.34 ± 0.06	1.25 ± 0.03	1.48 ± 0.01	0.15 ± 0.00	0.28 ± 0.00	0.06 ± 0.00	0.16 ± 0.00
	3 m³/h flow rate	0.35 ± 0.02	1.28 ± 0.04	1.42 ± 0.04	0.15 ± 0.01	0.27 ± 0.02	0.06 ± 0.00	0.15 ± 0.01
**Experiment**		**4-Nonyl-phenols (µg/L)**	**Lidocaine (µg/L)**	**Tramadol (µg/L)**	**Benzo-phenone (µg/L)**	**Diphenhy-dramine (µg/L)**	**TCEP (µg/L)**	**TCPP (µg/L)**
C	1 mg/L CC	1.31 ± 0.05	0.13 ± 0.01	0.23 ± 0.02	0.14 ± 0.02	0.20 ± 0.01	2.19 ± 0.24	1.18 ± 0.14
	3 mg/L CC	1.13 ± 0.03	0.15 ± 0.01	0.26 ± 0.02	0.12 ± 0.01	0.23 ± 0.01	0.37 ± 0.07	0.73 ± 0.04
	5 mg/L CC	1.60 ± 0.16	0.18 ± 0.01	0.14 ± 0.02	0.20 ± 0.01	0.16 ± 0.01	0.37 ± 0.01	1.36 ± 0.06
D	1 m³/h flow rate	0.03 ± 0.00	1.31 ± 0.03	0.14 ± 0.01	0.15 ± 0.03	0.28 ± 0.01	0.97 ± 0.06	1.22 ± 0.06
	3 m³/h flow rate	0.03 ± 0.00	1.04 ± 0.21	0.14 ± 0.00	0.19 ± 0.01	0.30 ± 0.02	0.70 ± 0.06	1.07 ± 0.06

B. Calculations

B.1. Second Order Kinetics

Two compounds A and B react with each other to form one or more compounds C:

$$A + B \rightarrow C \quad (1)$$

$$v = -\frac{d[A]}{dt} = -\frac{d[B]}{dt} = k[A][B] \quad (2)$$

The molar concentration of compound A at the time t ($[A]_t$) can be calculated with the knowledge of both initial molar concentrations $[A]_0$ and $[B]_0$ using the following equation:

$$[A]_t = \frac{[A]_0 \times e^{([A]_0 - [B]_0)kt} \times ([A]_0 - [B]_0)}{[A]_0 \times e^{([A]_0 - [B]_0)kt} - [B]_0} \quad (3)$$

The mass concentrations ϱ can be calculated using the molar masses M of the compounds:

$$\varrho(A) = [A] \times M(A); \varrho(B) = [B] \times M(B) \quad (4)$$

B.2. Inhibition Ratio of FAC by NH_4^+-N Between 0 and 1 mg/L NH_4^+-N

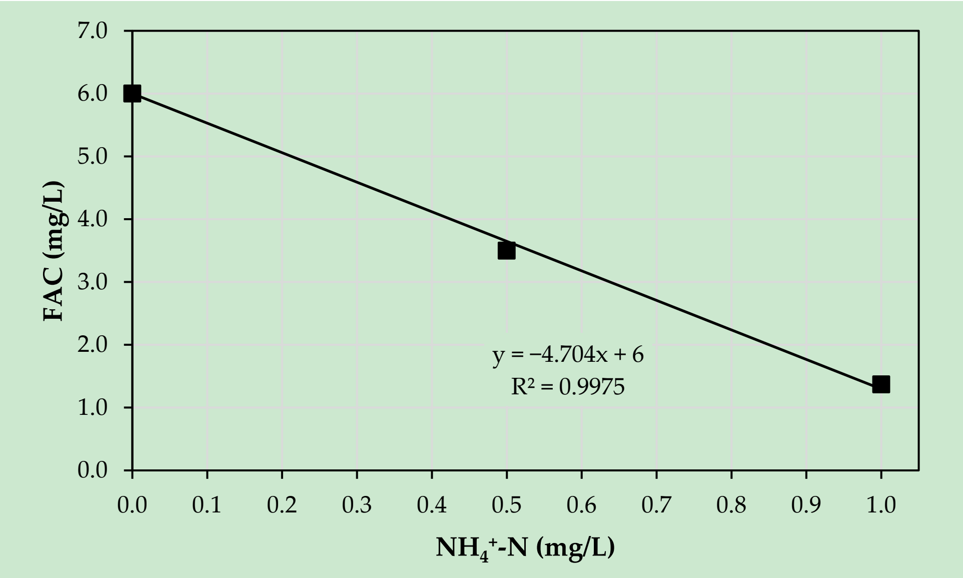

Figure S1. Measured FAC concentrations in the Cl_2 measuring cell as a function of the NH_4^+ concentration in the pilot plant influent in tap water matrix. The inhibition ratio is the slope of the function: 4.704 mg FAC per mg NH_4^+-N between 0 and 1 mg/L NH_4^+-N.

B.3. Determination of Contact Time Between Cl_2 Dosage Point and Cl_2 Measuring Cell

Reaction between: A: FAC (HOCl) and B: NH_4^+
Used rate constant: $k_{HOCl-NH_3} = 1.3\times10^4$ $M^{-1}s^{-1}$ (at pH 7)
Initial concentrations: $[A]_0 = 8.46\times10^{-5}$ M (= 6 mg/L FAC)
Initial concentrations: $[B]_0 = 0.00\times10^0$ M (= 0.0 mg/L NH_4^+-N)
$[B]_0 = 3.57\times10^{-5}$ M (= 0.5 mg/L NH_4^+-N)
$[B]_0 = 7.14\times10^{-5}$ M (= 1.0 mg/L NH_4^+-N)
$[B]_0 = 1.07\times10^{-4}$ M (= 1.5 mg/L NH_4^+-N)
Variation: t

Table S2. Results of least squares method to determine the contact time between Cl_2 dosage point and Cl_2 measuring cell.

NH_4^+-N measured	FAC calculated ($\varrho(A)_t$)					FAC measured
$\varrho(B)_0$ (mg/L)	$\varrho(A)_{0.500\ s}$ (mg/L)	$\varrho(A)_{1.500\ s}$ (mg/L)	$\varrho(A)_{3.500\ s}$ (mg/L)	$\varrho(A)_{5.605\ s}$ (mg/L)	$\varrho(A)_{6.122\ s}$ (mg/L)	$\varrho(A)_m$ (mg/L)
0.0	6.00	6.00	6.00	6.00	6.00	6.00
0.5	5.01	4.14	3.63	3.51	3.50	3.50
1.0	4.15	2.69	1.74	1.38	1.33	1.37
1.5	3.43	1.66	0.63	0.29	0.24	0.29
$\sum(\frac{\varrho(A)_t-\varrho(A)_m}{\varrho(A)_m})^2$:	121.515	23.19203	1.47238	0.00010	0.02763	-

With t = 5.605 s, the smallest possible sum of squares of the residuals could be achieved. The contact time between the Cl_2 dosage point and the Cl_2 measuring cell was therefore 5.6 s.

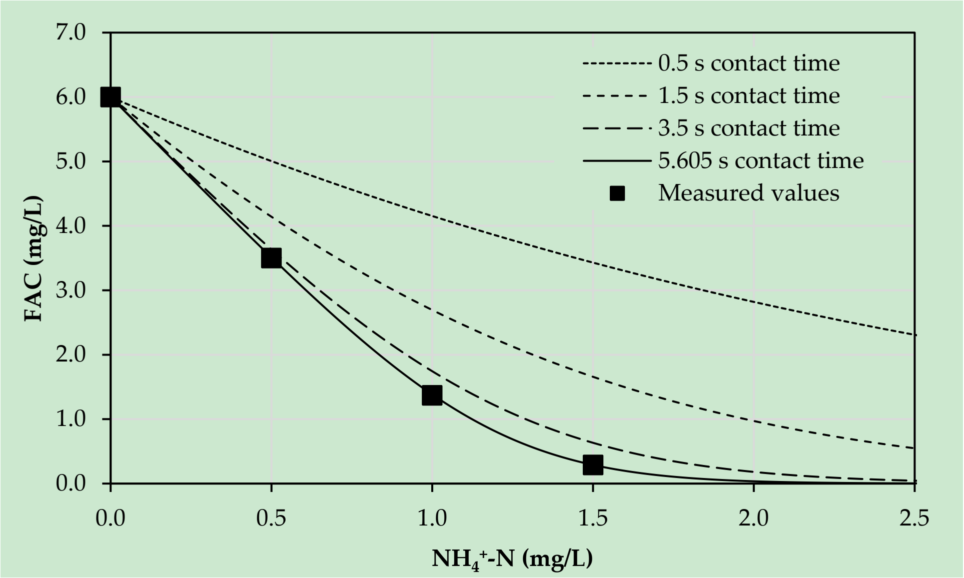

Figure S2. Results of least squares method to determine the contact time between Cl_2 dosage point and Cl_2 measuring cell. Approximation to measured data by changing the contact time at a fix second order rate constant: $k_{HOCl,NH_3} = 1.3\times10^4\ M^{-1}s^{-1}$.

B.4. Determination of Contact Time Between Cl_2 Dosage Point and UV Chamber Influent

The actual water volume between the Cl_2 dosage point and the UV chamber influent during the flow-through process is not equal to the pipe volume of this pipe section since the actual water volume of the static mixer is lower than the free available volume (considerable bubble formation). The water volume between the Cl_2 dosage point and the Cl_2 measuring cell consists of the water volume between the Cl_2 dosage point and the junction leading to the Cl_2 measuring cell (V_1) as well as the known pipe volume (104 cm^3) between this junction and the Cl_2 measuring cell (V_2). These volumes, the flow rate (F) and the contact time (t) can be combined using the Equations (5) and (6). With Equation (6), the water volume between the Cl_2 dosage point and the junction leading to the Cl_2 measuring cell (V_1) can be calculated.

$$V_1 + V_2 = F \times t \quad (5)$$

$$V_1 = F \times t - V_2 = 1\ m^3/h \times 5.605\ s - 104\ cm^3 = 1453\ cm^3 \quad (6)$$

With the knowledge of the water volume between the Cl_2 dosage point and the junction leading to the Cl_2 measuring cell (V_1) and the known pipe volume (247 cm^3) between this junction and the UV chamber influent (V_3), the contact time between the Cl_2 dosage point and the UV chamber influent can be calculated according to Table S3:

Table S3. Calculation of contact time between Cl_2 dosage point and UV chamber influent.

From Cl_2 dosage point to junction (to the Cl_2 measuring cell) (water volume V_1)	1453 cm^3
From junction (to the Cl_2 measuring cell) to the UV chamber influent (water volume = pipe volume) (V_3)	247 cm^3
From Cl_2 dosage point to the UV chamber influent (Σ water volume)	Σ 1700 cm^3
From Cl_2 dosage point to the UV chamber influent (Σ contact time)	1700 cm^3 / (1 m^3/h) = **6.122 s**

B.5. Inhibition Ratio of FAC by COD

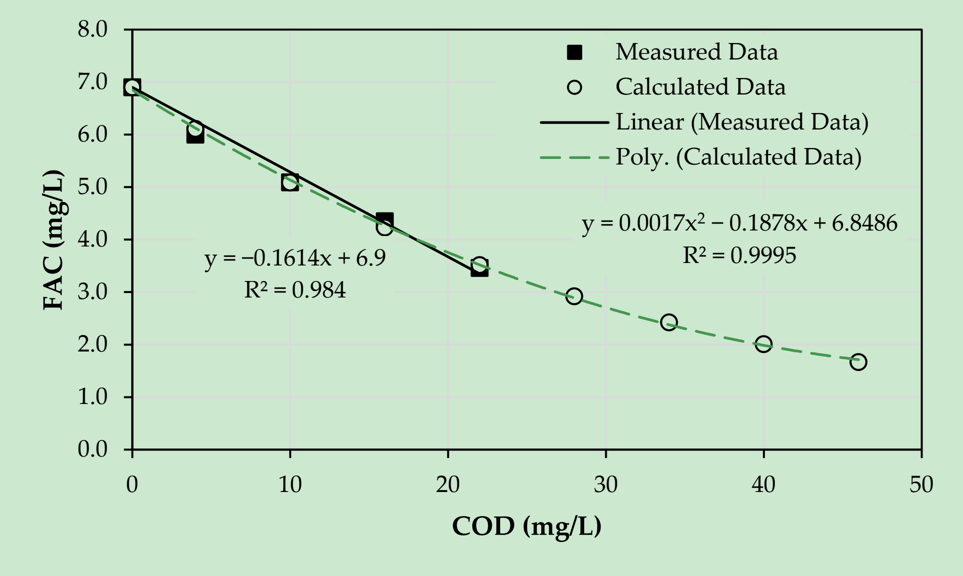

Figure S3. Measured FAC concentrations and calculated FAC concentrations (k = 182 $M^{-1}s^{-1}$) in the Cl_2 measuring cell as a function of the COD concentration in the pilot plant influent (dilutions of wastewater treatment plant effluent). The inhibition ratio between 0 and 22 mg/L COD can be assumed as the slope of the linear regression function gained from the measured data: 0.1614 mg FAC per mg COD. The inhibition ratio at COD concentrations >22 mg/L is not linearly proportional to the COD.

B.6. Determination of Rate Constant Between HOCl and COD

Reaction between: A: FAC (HOCl) and B: COD (O_2)
Used contact time: 5.605 s
Initial concentrations: $[A]_0 = 9.73\times10^{-5}$ M (= 6.9 mg/L FAC)
Initial concentrations: $[B]_0 = 1.25\times10^{-4}$ M (= 4 mg/L COD)
$[B]_0 = 3.13\times10^{-4}$ M (= 10 mg/L COD)
$[B]_0 = 5.00\times10^{-4}$ M (= 16 mg/L COD)
$[B]_0 = 6.88\times10^{-4}$ M (= 22 mg/L COD)
Variation: $k_{HOCl\text{–}COD}$

Table S4. Results of least squares method to determine the kinetic rate constant $k_{HOCl\text{–}COD}$.

$COD_{measured}$	$\varrho(A)_{t,calculated}$ at 5.605 s				$FAC_{measured}$	$\varrho(A)_{t,calculated}$ at 6.112 s
$\varrho(B)_0$ (mg/L)	$\varrho(A)_{50\ M^{-1}s^{-1}}$ (mg/L)	$\varrho(A)_{100\ M^{-1}s^{-1}}$ (mg/L)	$\varrho(A)_{150\ M^{-1}s^{-1}}$ (mg/L)	$\varrho(A)_{182.1\ M^{-1}s^{-1}}$ (mg/L)	$\varrho(A)_m$ (mg/L)	$\varrho(A)_{182.1\ M^{-1}s^{-1}}$ (mg/L)
4	6.67	6.44	6.24	6.11	6.00	6.04
10	6.33	5.82	5.36	5.09	5.09	4.95
16	6.01	5.25	4.60	4.23	4.35	4.05
22	5.70	4.74	3.95	3.52	3.46	3.31
$\sum\left(\frac{\varrho(A)_t-\varrho(A)_m}{\varrho(A)_m}\right)^2$:	0.63784	0.20483	0.02737	0.00135	-	-

With $k_{HOCl\text{–}COD}$ = 182.1 $M^{-1}s^{-1}$, the smallest possible sum of squares of the residuals could be achieved. The FAC concentration in the UV chamber influent (contact time of 6.112 s) differed by a maximum of 0.3 mg/L FAC from the FAC concentration in the Cl_2 measuring cell.

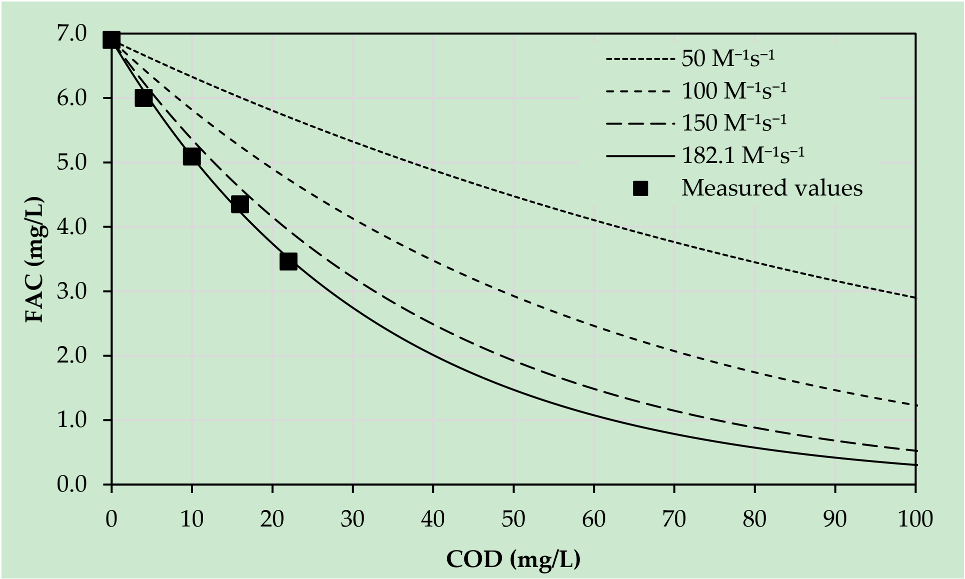

Figure S4. Results of least squares method to determine the kinetic rate constant $k_{HOCl-COD}$. Approximation to measured data by changing the kinetic rate constant at a fix contact time of 5.605 s.

B.7. Required Cl_2 Dosage to Obtain 3 mg/L FAC in the UV Chamber Influent

Table S5. Determination of regression curves to determine the required Cl_2 dosage for 3 mg/L FAC in the UV chamber influent as a function of NH_4^+-N or COD. A: FAC (HOCl). For regression functions, see Figure S5.

$\varrho(NH_4^+\text{-N})_0$ (mg/L)	$\varrho(A)_0$ calculated for $\varrho(A)_{6.112\,s}$=3 mg/L using k=1.3×10^4 M^{-1}s^{-1}	Values gained from linear regression of left column	Values gained from inhibition ratio	$\varrho(COD)_0$ (mg/L)	$\varrho(A)_0$ calculated for $\varrho(A)_{6.112\,s}$=3 mg/L using k=182.1 M^{-1}s^{-1}	Values gained from polynomial regression of left column	Values gained from inhibition ratio
0.0	3.00	3.00	3.00	0	3.00	3.00	3.00
0.5	5.48	5.52	5.35	5	3.55	3.55	3.81
1.0	7.99	8.04	7.70	10	4.20	4.19	4.61
1.5	10.51	10.56	10.06	20	5.86	5.85	6.23
2.0	13.03	13.07	12.41	30	8.10	8.12	7.84
3.0	18.09	18.11	17,11	40	11.12	11.15	9.46
5.0	28.21	28.19	26.52	50	15.10	15.11	11.07
10.0	53.51	53.37	50.04	60	20.15	20.15	12.68
15.0	78.82	78.56	73.56	70	26.50	26.42	14.30
20.0	104.13	103.74	97.08	80	34.20	34.09	15.91
25.0	129.44	128.93	120.60	90	43.40	43.29	17.53
				100	53.90	54.20	19.14

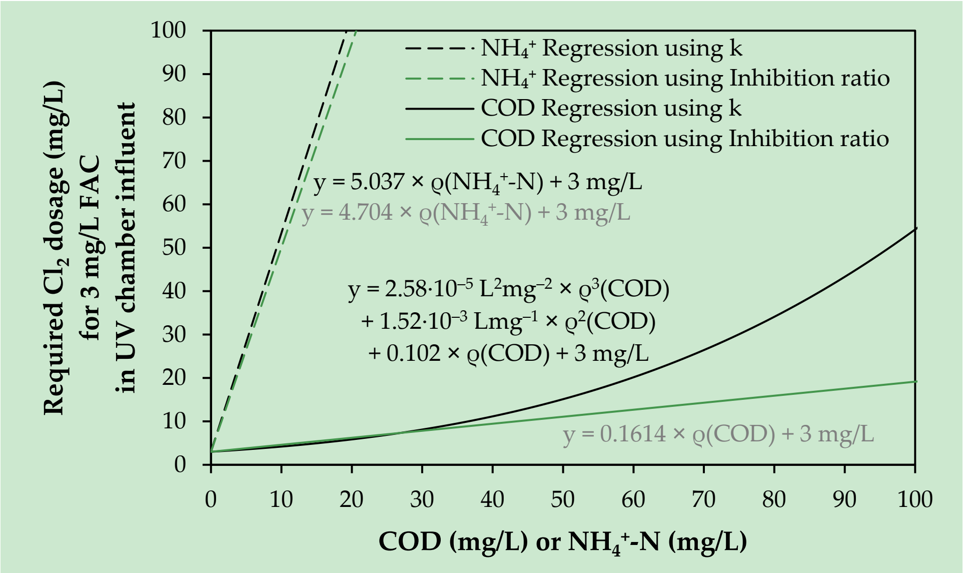

Figure S5. Required Cl_2 dosage to obtain 3 mg/L FAC in the UV chamber influent (6.112 s contact time) in the presence either of ammonium ions or COD (the results may differ when both are present at the same time) calculated either using the kinetic rate constants (k_{HOCl,NH_3} = 1.3×10^4 M^{-1}s^{-1} and $k_{HOCl-COD}$ = 182.1 M^{-1}s^{-1}) or the inhibition ratios (4.7 mg FAC per mg NH_4^+-N and 0.16 mg FAC per mg COD).

B.8. Electrical Energy Consumption per Order of Compound Removal

Table S6. Electrical energy consumption per order of compound removal E_{EO} as calculated from the data given in Figure 8.

NH_4^+-N (mg/L)	COD (mg/L)	$E_{EO, carbamazepine}$ (kWh/m^3/order)	$E_{EO, diclofenac}$ (kWh/m^3/order)
0.0	<5	0.51	0.18
0.5	<5	0.72	0.41
1.0	<5	1.15	0.49
1.5	<5	2.23	0.54
<0.1	10	0.79	0.29
<0.1	16	1.16	0.39
<0.1	22	2.23	0.47

Table S7. Electrical energy consumption per order of compound removal E_{EO} (kWh/m³/order) as calculated from the data given in Figure 9.

	CC	UV/CC	UV/FAC	CC	UV/CC	UV/FAC	CC	UV/CC	UV/FAC	CC	UV/CC	UV/FAC
Oxidant	Carbamazepine			Diclofenac			Bisphenol A			AHTN		
0 mg/L	3.65	3.65	3.65	0.55	0.55	0.55	2.69	2.69	2.69	1.07	1.07	1.07
1 mg/L	-13.56	16.59	2.04	8.11	2.30	0.56	58.58	2.95	0.82	32.79	1.27	0.62
3 mg/L	25.34	5.97	1.70	-19.93	1.04	0.46	-39.00	2.31	0.51	34.56	1.18	0.57
5 mg/L	-16.34	10.36	1.41	4.61	0.48	0.32	62.62	2.47	0.47	-84.96	0.97	0.53
Oxidant	4-Nonylphenols			Tramadol			Diphenhydramine			HHCB-lactone		
0 mg/L	1.87	1.87	1.87	1.25	1.25	1.25	3.46	3.46	3.46	7.87	7.87	7.87
1 mg/L	-12.29	4.40	1.00	-56.28	4.57	1.34	11.93	4.46	0.88	105.40	6.72	1.71
3 mg/L	9.49	7.05	0.70	-33.47	2.45	0.80	11.18	3.60	0.62	-37.45	4.84	1.05
5 mg/L	199.76	1.59	0.51	6.03	2.62	0.75	10.23	3.02	0.55	38.07	1.58	0.80
Oxidant	Benzophenone			MTBT			HHCB			Lidocaine		
0 mg/L	11.37	11.37	11.37	2.62	2.62	2.62	3.73	3.73	3.73	4.04	4.04	4.04
1 mg/L	17.42	-13.02	3.16	14.27	4.92	2.39	353.78	4.77	2.67	-10.84	-23.43	2.08
3 mg/L	11.83	14.83	1.84	4.44	2.63	1.36	40.66	3.21	1.63	15.09	7.02	1.61
5 mg/L	81.77	1.90	0.97	11.88	1.38	0.96	21.26	1.52	1.38	-24.57	16.82	1.30
Oxidant	4t-Octylphenol			DEET			TCPP			TCEP		
0 mg/L	-8.80	-8.80	-8.80	2.19	2.19	2.19	4.70	4.70	4.70	4.39	4.39	4.39
1 mg/L	-26.48	-5.90	3.28	117.62	11.88	4.08	17.05	-37.60	11.81	14.83	-20.39	-37.30
3 mg/L	-87.35	-124.92	1.47	-7.04	-318.06	3.13	-7.37	4.80	-184.67	38.40	-20.10	-11.83
5 mg/L	-23.26	6.00	0.93	41.79	1.88	2.47	24.03	1.77	16.28	40.29	1.57	-46.51

Table S8. Electrical energy consumption per order of compound removal E_{EO} (kWh/m³/order) as calculated from the data given in Figure 10.

kWh/m³	Carbamazepine	Diclofenac	Bisphenol A	AHTN	4-Nonylphenols	Tramadol	Diphenhydramine	HHCB-lactone
0.00	49.59	1.24	0.72	30.34	0.90	2.06	0.88	-84.19
0.13	2.47	0.53	0.47	1.02	0.79	1.22	0.69	1.33
0.20	2.01	0.42	0.44	0.77	0.72	0.91	0.68	1.07
0.40	1.26	0.37	0.45	0.55	0.68	0.97	0.75	0.86
0.70	1.29	0.36	0.41	0.43	0.55	0.57	0.60	0.75
1.00	1.18	0.33	0.40	0.40	0.52	0.55	0.60	0.70
kWh/m³	Benzophenone	MTBT	HHCB	Lidocaine	4t-Octylphenol	DEET	TCPP	TCEP
0.00	8.24	2.08	30.76	11.39	1.11	42.18	10.83	41.03
0.13	2.33	2.00	2.22	2.25	1.29	5.22	-26.26	-11.69
0.20	1.59	1.49	1.85	1.91	1.32	2.78	16.59	14.29
0.40	1.05	1.10	1.41	1.36	1.07	1.73	7.44	13.66
0.70	0.83	1.02	1.24	1.34	1.54	1.68	6.10	8.33
1.00	0.79	0.96	1.12	1.38	1.40	1.32	5.18	4.88
